# Novel computer-aided reconstruction of soft tissue defects following resection of oral and *oropharyngeal* squamous cell carcinoma

**DOI:** 10.1186/s12957-022-02654-7

**Published:** 2022-06-13

**Authors:** Jiajie Xu, Fangyuan Lai, Yunfeng Liu, Zhuo Tan, Chuanming Zheng, Jiafeng Wang, Haiwei Guo, Liehao Jiang, Xinyang Ge, Xiabin Lan, Chao Chen, Minghua Ge

**Affiliations:** 1 Otolaryngology& Head and Neck Center, Cancer Center, Department of Head and Neck Surgery, Zhejiang Provincial People’s Hospital (Affiliated People’s Hospital, Hangzhou Medical College), 310014 Hangzhou, China; 2 Key Laboratory of Endocrine Gland Diseases of Zhejiang Province, Hangzhou, 310014 China; 3 Center for Plastic & Reconstructive Surgery, Department of Plastic & Reconstructive Surgery, Zhejiang Provincial People’s Hospital (Affiliated People’s Hospital, Hangzhou Medical College), 310014 Hangzhou, China; 4 Key Laboratory of E&M, Zhejiang University of Technology, Ministry of Education & Zhejiang Province, Hangzhou, 310014 China; 5 College of Mechanical Engineering, Zhejiang University of Technology, Ministry of Education & Zhejiang Province, Hangzhou, 310023 China; 6 College of Letters and Science, University of California, Los Angeles, Los Angeles, CA 90095 USA; 7 Department of Head and Neck Surgery, Cancer Hospital of the University of Chinese Academy of Sciences, Zhejiang Cancer Hospital, Hangzhou, 310022 China

**Keywords:** Oral and oropharyngeal, Tongue, Squamous cell carcinoma (SCC), Anterolateral thigh flap (ALTF), Computer-aided reconstruction of soft tissue (CARST)

## Abstract

**Background:**

Reconstruction of soft tissue defects following surgical tumor resection is important for quality of life in cancer patients with oral and oropharyngeal squamous cell carcinoma (SCC). This study presents a novel computer-aided reconstruction of soft tissue (CARST) technology employed with these patients.

**Methods:**

We first described the CARST technology in detail in a report of a 34-year-old male patient with locally invasive right-sided tongue SCC following a nearly total glossectomy and reported the postoperative outcomes. This digital technology was applied to construct a 3D model from CT images, which was used to delineate surgical resection boundaries and design a personalized reconstruction of the soft tissue defect. A nonuniform rational B-spline (NURBS) was generated and applied to transform the 3D model into a 2D flap-cutting guide printed out using a 3D printer. We then reported a case-series study on oral and oropharyngeal SCC patients who were randomly assigned to receive the CARST (*n* = 15) or a traditional soft tissue reconstruction (*n* = 15). Clinicopathological features and short- and long-term postoperative outcomes between the two groups were compared.

**Results:**

The patient with the tongue SCC had a successful CARST following surgical tumor resection without any complications. His speech and swallowing functions recovered well after surgery and he experienced no significant changes to his appearance following recovery. There was no recurrence within a 3-year follow-up period. Results of the case-series study showed that the CARST group had significantly shorter operative and post-operation hospital-stay time, a higher flap utilization rate, and a trend of less and milder postoperative complications, and they experienced no significant difference in intraoperative blood loss and long-term outcomes compared to the traditional group.

**Conclusion:**

CARST is a safer and more efficient personalized technology of soft tissue reconstruction following surgical tumor resection in patients with oral and oropharyngeal SCC.

**Supplementary Information:**

The online version contains supplementary material available at 10.1186/s12957-022-02654-7.

## Introduction

Oral and oropharyngeal squamous cell carcinomas (SCC) are a group of malignancies that comprise the majority of head and neck cancers [1], while tongue SCC is common cancer diagnosed within the oral cavity [2]. Although oral and oropharyngeal cancer can be detected early, patients are frequently diagnosed in advanced stages. Surgical resection is a key component of multidisciplinary approaches in the treatment of oral and oropharyngeal SCC [3]. Both the tumor itself and the extensive surgical resection can cause significant and complex defects in the tongue body, mouth floor, and oropharynx, subsequently affecting speech and swallowing functions. Reconstruction of soft tissue and restoration of tongue function following surgical tumor resection is important for quality of life in these cancer patients. In traditional surgical treatment, doctors often rely on their own clinical experience for tumor resection, skin flap preparation, and soft tissue defect reconstruction. Due to the complex anatomical structures and special functions of affected sites, it is challenging to achieve precise preparation of the skin flap and reconstruction of soft tissue defect, and to accomplish ideal aesthetic and functional outcomes.

The application of appropriate flaps is crucial to reconstruct composite soft tissue defects following surgical resection of oral and oropharyngeal cancer. Both pedicled flap and free flap have been applied in the reconstruction of soft tissue following surgical removal of tongue cancer. The pedicled flaps include the adjacent local flap (submental island flap) and the distal pedicled flap (pectoralis major musculocutaneous flap). The free flaps include the radial forearm free flap (RFFF), the anterolateral thigh flap (ALTF), the deep inferior epigastric artery perforator flap and inferior rectus abdominis flap [4–6]. The disadvantages of using the submental island flap are tissue insufficiency and a high risk of pedicle torsion and necrosis [4–7]. Resection of the pectoralis major myocutaneous flap may result in a bulky structure and severe wounding at the donor site. With the progress of microsurgical techniques, utilization of free flaps, particularly the RFFF and ALTF, has become common in soft tissue reconstruction. The RFFF is soft, thin and easy to fold. Due to the limited quantity of this tissue, it is suitable for surgical defects that comprise less than half of the tongue body [4]. When the surgical defect is large, and especially when it exceeds two-thirds of the tongue body, the ALTF is usually selected due to its great flexibility and abundance of pliable soft tissue and large-caliber vascular pedicle, and this results in less damage to donor sites [8]. The chimeric perforator flaps, which consist of multiple cutaneous territories, are a new alternative for significant defects of the mouth floor when a single perforator flap may not achieve a satisfactory result [9].

Digital technology has been increasingly applied in assisting in mandibular bone reconstruction following surgical tumor resection with satisfactory outcomes [10–15]. Due to the complex surfaces of the head, neck, face, and tongue, and the elasticity of soft tissue and irregular spatial structure, very few studies have applied digital technology in the reconstruction of soft tissue defects [16]. Herein, we describe the CARST technology in detail following surgical resection in a patient with locally advanced tongue SCC. We then compared clinical outcomes in other oral and oropharyngeal cancer patients who underwent radical resection with or without CARST. This novel technology helps surgeons to formulate a personalized surgical plan and gains clinically significant benefits.

## Patients and methods

### Case presentation

The 34-year-old male patient visited the Department of Head and Neck Surgical Oncology of our hospital on September 25, 2017. The patient himself found a right tongue ulcer with paroxysmal pain with no bleeding or dysphagia 2 months prior. It was then diagnosed as “tongue ulcer” and treated with anti-inflammatory drugs in his local hospital. Due to deteriorating symptoms, including gradually enlarged ulcerative mass in the right tongue and an aggravating pain, the patient was referred to our department for further diagnosis and treatment for suspected tongue cancer.

#### Physical examination

The patient’s mouth opening was normal, but his tongue had limited movement. A visible ulcerative tumor of 4.0 cm in diameter was found on the right tongue. The tumor had a hard texture with obvious tenderness showing an infiltrative growth pattern with blurred boundaries. The tumor invaded into the left side of the tongue, and involved the right tongue base and mouth floor. Lymphadenectases of up to approximately 2.0 cm in diameter were found on the right mandible and right upper neck. These nodes had moderate texture, normal mobility with no tenderness.

#### Oral and oropharyngeal enhanced CT scans

The tumor was shown as a dense shadow (2.0 cm × 4.4 cm × 2.5 cm) with blurred boundaries and an ulcer on its surface that became clear after the enhancement. The lesion invaded upward to tongue back (Supplemental data Figure S1A), downward to ventral tongue and mouth floor (Supplemental data Figure S1B), inward beyond the midline to contralateral side (Supplemental data Figure S1C), outward to the right edge of the tongue (Supplemental data Figure S1D), forward to one-third of the tongue body (Supplemental data Figure S1E) and backward to tongue base (Supplemental data Figure S1F). Tongue tip, oropharyngeal wall and adjacent bones were not involved. The pathological result on needle biopsy reported “(tongue) well-differentiated squamous cell carcinoma”.

#### Computer-aided reconstruction of soft tissue (CARST)

**Fig. 1:**
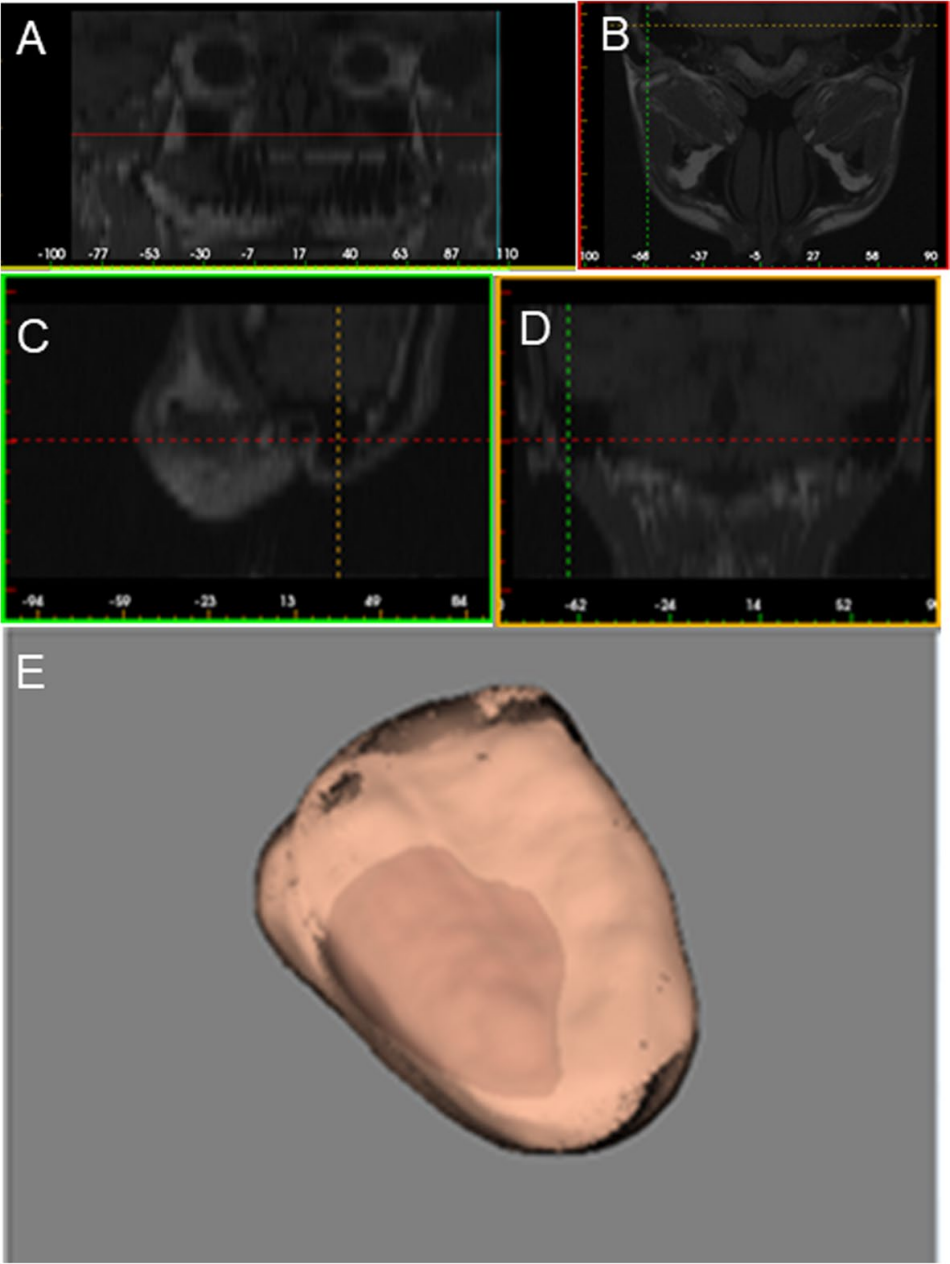
Generating a model of the tumor and the affected soft tissue. **A** Thin layer CT scan of curved planar reconstruction panorama. **B** Horizontal CT scan. **C** Sagittal CT scan. **D** Coronal CT scan. **E** 3D .stl model

**Fig. 2:**
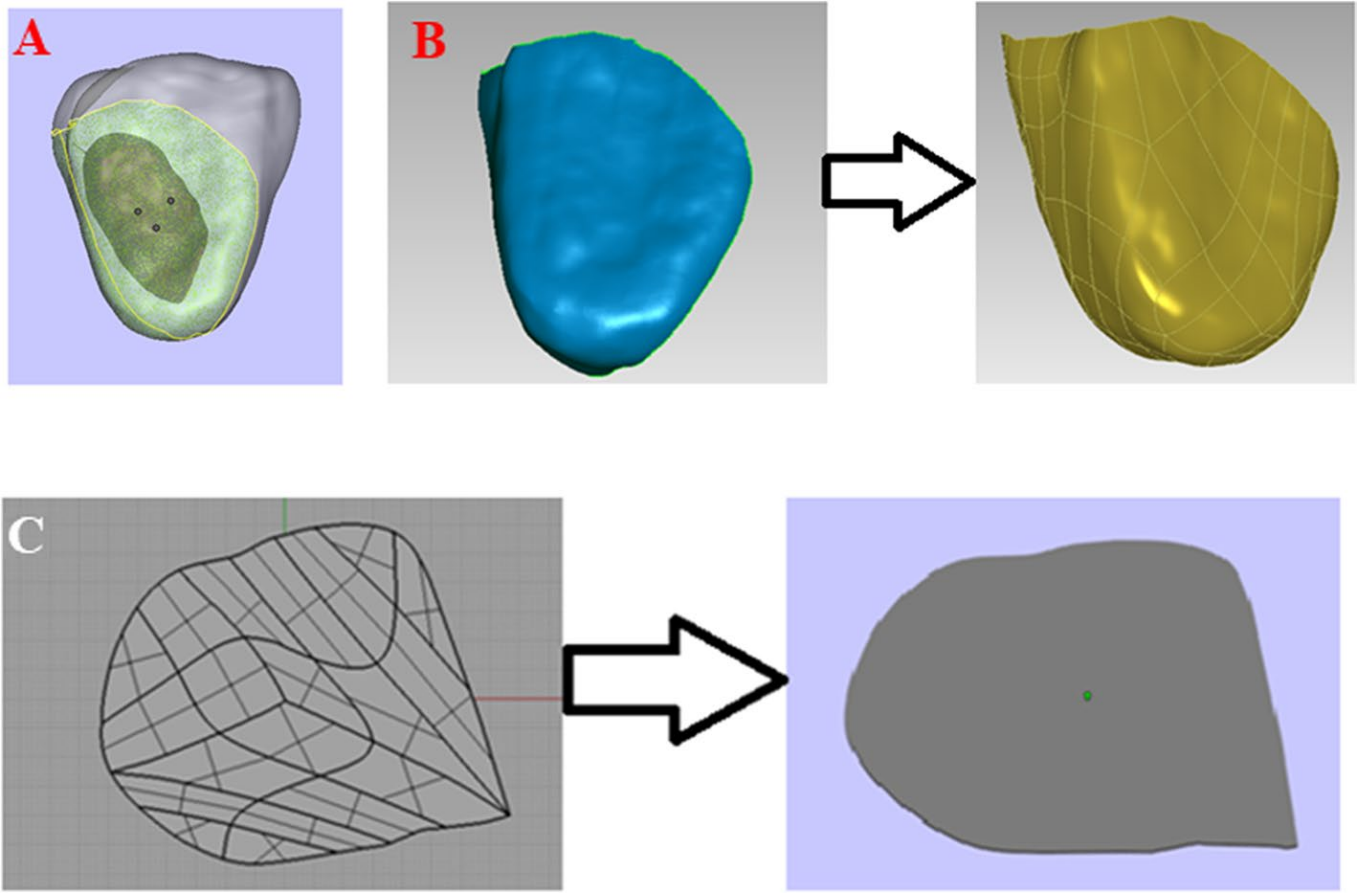
The application of CARST. **A** Virtual safe resection boundary. **B** Convert curved surface of the soft-tissue to be reconstructed to NURBS curved surface. **C** The 3D model was transformed into a 2D flap cutting guide

The patient’s thin-layer (0.2 mm per layer) CT images were converted into the DICOM format in order to be imported into the 3D modeling software (6D-DENTAL Tech Co, Ltd., China). A 3D model of the tumor and surrounding soft tissue was generated as a stereolithography (stl) file (Fig. 1A–E). Using the software packages of Magics (Materialise Ltd., the Kingdom of Belgium) and Geomagice Studio (Geomagice, USA), this 3D model was further used to demarcate the resection boundaries based on the safe resection range (around 2.0 cm from the tumor border) and design the personalized reconstruction of soft tissue (Fig. 2A). A nonuniform rational B-spline (NURBS) was then generated for the 3D model using Geomagice studio software (Fig. 2b). Based on the curvature and stretchability, the NURBS was adjusted and flattened to generate a 2D flap cutting guide using Rhino software (Robert McNeel Ltd., USA), which was printed out on a Connex 350 3D printer (Stratasys ltd, USA) using a photosensitive resin (Objet MED 610, Objet Ltd., Israel) (Fig. 2C).

The surgery was performed under general anesthesia on October 10, 2017. The intraoperative exploration revealed that the tumor had a hard texture and blurred boundaries with a size of approximately 4.0 × 3.0 cm which was mainly located on the right tongue. The right tongue base and oropharyngeal side wall were involved. The tumor body crossed the tongue midline to the left side. Lymphadenectasis was found bilaterally in the neck. The large lymphadenectasis lesions were located in the right upper neck with moderately hard texture and a size of approximately 2.0 × 1.5 cm.

**Fig. 3:**
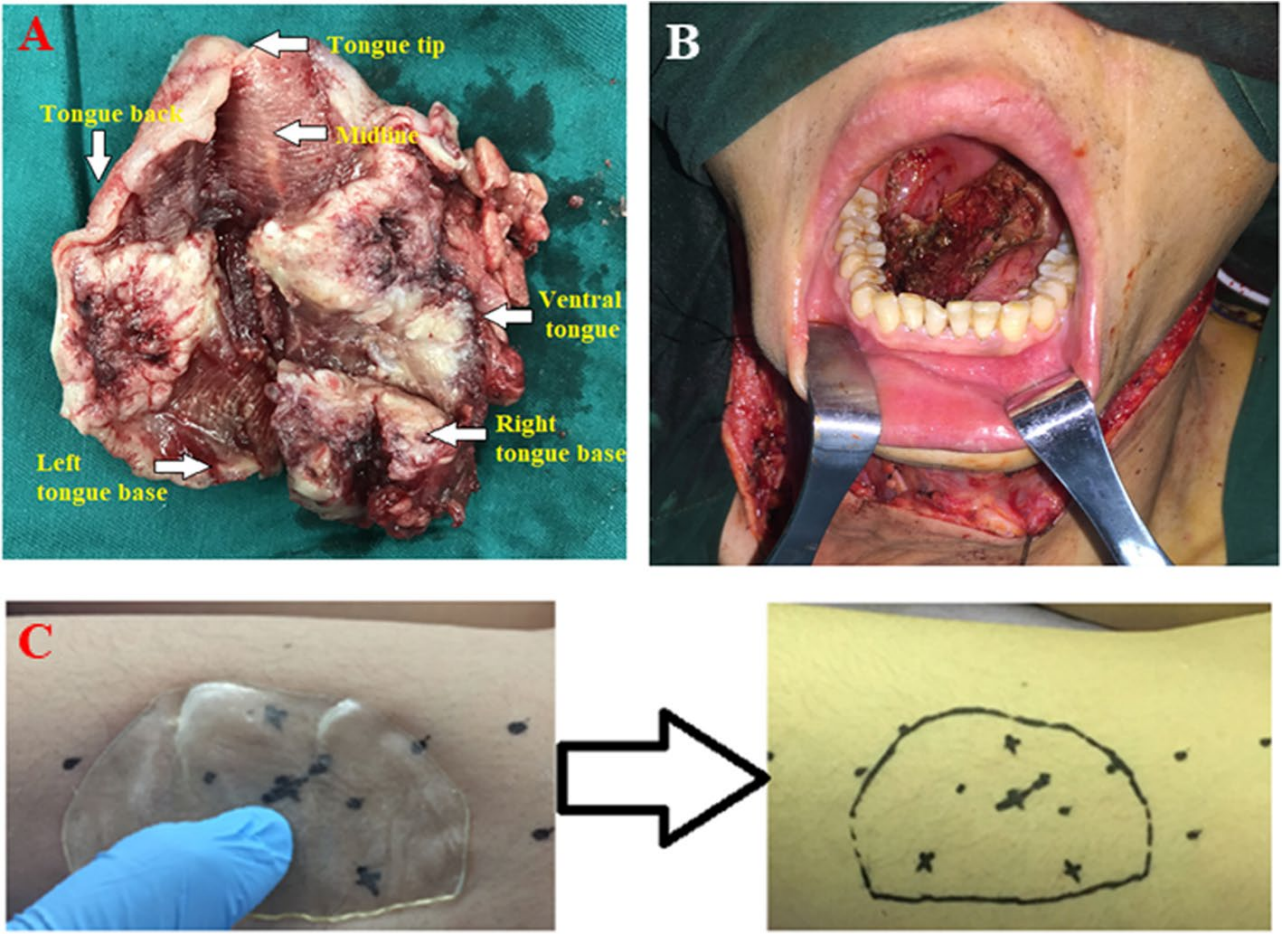
Surgical resection of the tumor and flap preparation. **A** The resected tumor. **B** Retained left tongue base. **C** Flap perforator blood vessels were located by preoperative Doppler ultrasound and flap perforator was marked with “X”. The surface area was defined by the 3D-printed flap cutting guide plate and with the “X” as its center

One group of surgeons completed lymph node dissection in the I~V areas of the right neck and the I~III areas of the left neck, and then resected the entire tumor with the safe resection boundary designed preoperatively (Fig. 3A). Approximately, threequarters of the tongue was removed while the left tongue base was retained (Fig. 3B). Pathological diagnosis on the intraoperative frozen slices confirmed negative resection margins.

**Fig. 4:**
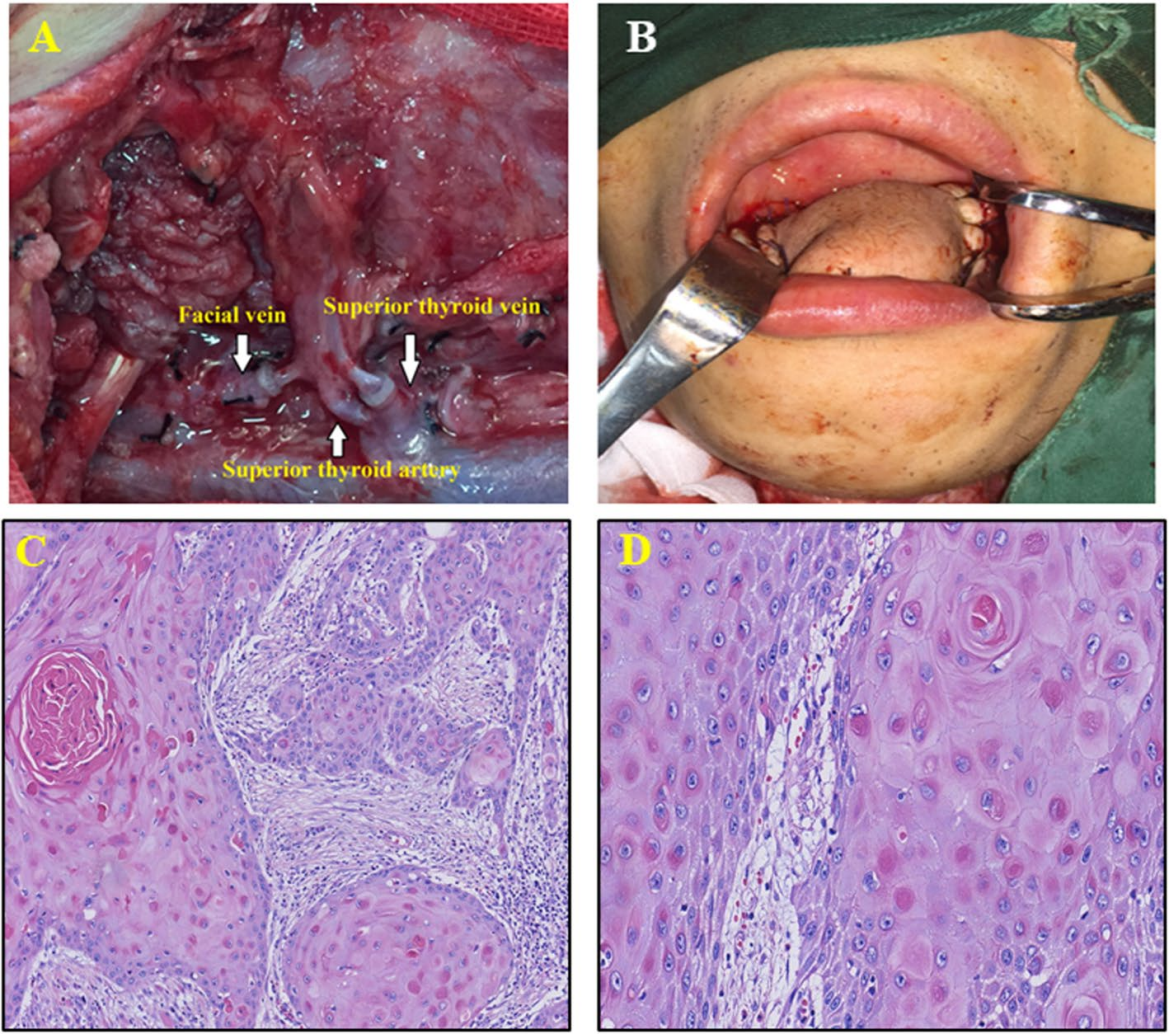
Blood vessels for anastomoses, reconstructed tongue and pathological images. **A** Descending branch of lateral circumflex femoral artery was anastomosed with right superior thyroid artery, and venae comites were anastomosed with right facial vein and right superior thyroid vein. **B** The flap was sutured to the defect area on the oral cavity and right oropharynx to create a tongue-like shape. **C** H&E, × 100, infiltrative growth and arranged in a nested pattern. **D** H&E, × 200, single cell keratinization and intercellular bridge

Meanwhile, a second group of surgeons prepared the flap at the left thigh. A longitudinal incision was performed along the left thigh. Flap perforator blood vessels were located by preoperative Doppler ultrasound and marked with an “X”. The surface area was defined by the cutting guide plate described above with the “X” as its center (Fig. 3C). During the surgical cutting, the descending branch of the lateral circumflex femoral artery and the venae comites were dissected. The pedicle was cut and rinsed with anticoagulant buffer. The descending branch of lateral circumflex femoral artery was anastomosed with the right superior thyroid artery, and the venae comites was anastomosed with the right facial vein to assure smooth blood flow and enough blood supply for the edge of the flap (Fig. 4A). The surgeons sutured the flap to the defect area on the oral cavity and right oropharynx to form a tongue-like shape (Fig. 4B). A drainage tube was placed and the incision was sutured. The trachea was incised. A trachea cannula was inserted and connected to auxiliary ventilation and sputum drainage. The patient was monitored in an intensive care unit (ICU) for 24 h after the operation.

#### Postoperative pathological results

The tumor exhibited infiltrative growth in a nested pattern. Cellular keratinization, including both keratin pearl and single cell keratinization, was observed in the lesion. Intercellular bridges could be seen between adjacent cancer cells. Nuclei showed atypia with visible nucleoli (Fig. 4C, H&E, × 100 times). Higher magnification clearly identified single cell keratinization and intercellular bridges (Fig. 4D, H&E, × 200).

#### Short- and longterm outcome

The flap was in good condition with no necrosis after the surgery. Occlusion and mouth opening were normal. No significant change was observed in facial appearance (Supplemental data Figure S2A–S2H). The size of the rebuilt tongue was suitable for normal functions of chewing and swallowing. Speech ability was partially impaired. The patient was discharged from the hospital 14 days after the surgery. One-month post-operative CT scan indicated that the ALTF healed well with the surrounding tissue and no tumor residues were found (Supplemental data Figure S3A–S3F). He was able to take fluid and semi-liquid food by mouth 2 weeks after surgery, and eat various foods including meat and nuts even after undergoing radiotherapy 3 months after surgery. The patient was still alive with improved speech and without recurrence over 3 years after the surgery. There was no damage to the tongue on either side. The patient had almost normal tongue functions, facial appearance, and a highly satisfactory quality of life.

### Review of case series

During January 2018–June 2020, we randomly assigned 15 patients with oral and oropharyngeal SCC to receive CARST and another 15 patients underwent traditional soft tissue reconstruction following surgical resection. The clinicopathological features and postoperative outcomes, including success rate, efficiency of flap use, surgical time, and perioperative complications, were compared between the CARST and traditional reconstruction groups.

### Data analysis

Due to the limited sample size, both the categorical and continuous data were analyzed using Fisher’s exact test. A two-sided *p* < 0.05 was defined as statistically significant. The SAS software V9.3 (SAS *Institute*, Inc., Cary, NC, USA) was used for the data analysis.

## Results

The clinicopathologic features of oral and *oropharyngeal* SCC patients in the CARST and the traditional groups are presented in Table 1. There were no significant differences in patients’ ages, ratio of male to female, primary sites of cancer, and TNM staging between the two groups.

**Table 1:** Clinicopathologic characteristics of the patients in the CARST and traditional groups

Characteristics	CARST group (*n* =1 5)	Traditional group (*n* = 15)	*P* value
Sex			
Male	12	10	0.68
Female	3	5	
Age (years)	65.9 ± 7.5	62.6 ± 10.8	0.24
Primary tumor location			1.00*.*
Tongue	6	6	
Tonsil base	2	2	
Buccal mucosa	2	2	
Floor of mouth	1	1	
Palate	2	2	
Tongue base	2	2	
Pathological type	Squamous cell carcinoma	Squamous cell carcinoma	
Tumor (topography)			0.55
T1	0	0	
T2	1	0	
T3	12	12	
T4	2	3	
Lymph node			0.64
N0	4	2	
N1	3	3	
N2	8	10	
N3	0	0	
Metastasis			1.00
M0	15	15	
M1	0	0	
TNM stage			0.41
I	0	0	
II	0	0	
III	5	3	
IVa	10	12	

Patients in the CARST group had significantly shorter operative time (366 ± 71.7 vs 411.3 ± 74.1 min, *P* = 0.01), a higher flap utilization rate (82.9 ± 6.5 vs 66.5 ± 9.0, *P* = 0.001) and reduced post-operative hospital stay time (13.7 ± 2.7 vs 16.2 ± 3.3 days, *P* = 0.03) compared to the traditional group. Patients in both groups had similar intraoperative blood loss and amounts of drainage (Table 2). In addition, patients in the two groups had comparable postoperative complications. In the CARST group, 4 cases had postoperative complications, including 1 venous crisis, 1 seroma, and 2 postoperative infections. Postoperative complications occurred in 8 cases of the traditional reconstruction group, which included 2 venous crises, 1 seroma, 4 postoperative infections, and 2 focal necroses of the flap (including one with both infection and focal necrosis). Except for those with infections, all patients were readmitted for surgical treatments due to complications (Table 3). Though the results were not significantly different, the CARST group tended to have fewer and milder complications.

**Table 2:** Comparison of surgical outcomes between the CARST and traditional groups

Outcome	CARST group (*n* = 15)	Traditional group (*n* = 15)	*P* value
Operative time (min)	366 ± 71.7	411.3 ± 74.1	0.01
Intraoperative blood loss (ml)	370.7 ± 80.0	376.0 ± 65.2	0.81
Amount of drainage (ml)	476.7 ± 77.8	514.0 ± 93.0	0.09
Flap utilization rate (%)	82.9 ± 6.5	66.5 ± 9.0	0.00
Post-operational hospital stay (days)	13.7 ± 2.7	16.2 ± 3.3	0.03

**Table 3:** Comparison of postoperative complications between the CARST and traditional groups

Complication	CARST group (*n* = 15)	Traditional group (*n* = 15)	*P* value
Post-operative complication			
No	11	7	0.26
Yes	4	8	
Type of complications			0.13
Local necrosis	1	2	
Total necrosis	0	0	
Venous crisis	1	2	
Arterial crisis	0	0	
Seroma	1	1	
Infection	2	4	

After a median follow-up time of 19 months (range: 7–36 months), there were no recurrences or deaths among all these patients.

## Discussion

Our results indicate that it is safer and more efficient to utilize the novel CARST following resection of oral and oropharyngeal cancer. The tongue SCC patient was able to take fluid and semi-liquid food by mouth 2 weeks after surgery. Even though the patient received radiotherapy 3 months later, he was able to eat various foods including meat and nuts. The patient retained maximum tongue function and normal facial appearance with a highly satisfactory quality of life. Our data further indicate that the novel CARST can also be applied in the reconstruction of soft tissue defects after resection of primary cancers in the floor of the mouth, tonsil, buccal, palate, and tongue base. The new technology significantly reduced the operative time and possibly hospital stay time and improved the utilization rate of the flap.

Surgical resection of primary oral and oropharyngeal SCC and surrounding tissues or organs frequently causes defects of the tongue, tongue base, mouth floor, lateral wall of oropharynx, mandible, and larynx. Reconstruction of soft tissue defects following surgical resection requires a multidisciplinary team including oncologic surgeons, plastic surgeons and even digital technology scientists. The CARST digital technology enables teams to build a 3D model to formulate a personalized surgical plan that delineates clear and safe surgical boundaries, provides the design for reconstruction of the soft tissue defect and produces the flap cutting guide. Our data showed that the novel CARST technology can also be applied to soft tissue defect repair caused by cancer or other diseases in head, neck and other sites.

Due to the special role of the tongue in the oral cavity, reconstruction of the tongue should allow for restoration of speech, deglutition, and other functions. The re-establishment of tongue dynamic function after total or near total glossectomy is still a significant challenge. Lift of the reconstructed tongue can only be achieved through coordination of the residual suprahyoid muscles and parapharyngeal muscles. It is vital to retain as much functional adjacent soft tissue as possible under the premise of negative tumor resection margin. The novel CARST aids in delineating safe surgical boundaries and thus improves the chance of fully restoring the dynamic function of the tongue. CARST technology reduces waste of flap tissue, operative time and possibly hospital stay time.

Both pedicled flap and free flap have been applied to repair tongue defects after tumor resection. It is currently believed that the use of free flaps to repair defects of the tongue and its adjacent tissue has the advantage of providing more comfort and desirable aesthetics compared to the use of pedicle flaps [17]. The ALTF has been widely used in the reconstruction of oral, and head and neck soft tissue defects [18, 19] and a number of improvements have been made [20]. Results from our study support the use of ALTF in soft tissue reconstruction.

Donor-site morbidities for preparing flaps are reported in up to 53% of cases including tendon exposure. These include delayed healing, reoperation, tendon adhesion, limitation of joint movement, and poor cosmesis [21]. Minimizing damage to the donor area is an important part of the successful treatment of head and neck defects. A previous study suggests that a change in the flap design for a proximal site along the forearm or suprafascial elevation can be useful to decrease donor site morbidities, such as pain and scarring [22]. Our novel CARST strategy minimized the damage to the donor site by precisely estimating the size and shape of the flap and improving the flap utilization rate. Recent studies have reported improved outcomes for donor-site morbidity by employing dermal substitutes, tissue expander, local flap with rotation, and full thickness skin grafts [9, 10, 13, 15, 23, 24].

A recent study described application of a similar digital technology, Personalised pAtient-specific plaNning of SOFt tissue reconstruction *(*PANSOFOS), following surgical resection of the tongue SCC [16]. The 3D models for the reconstructed soft tissue defect were built from the CT images and further developed for a flap cutting guide. Both this study and ours demonstrated excellent oncological, functional and cosmetic outcomes of the digital technology-assisted and patient-specific soft tissue reconstruction in patients with oral and oropharyngeal SCC. In the PANSOFOS study, a 3D mold was directly fabricated by a 3D printer and then filled with silicone impression material which was transformed into a 2D flattened shape that was used as the flap cutting guide. In our study, the 3D model was used to generate NURBS, then was digitally transformed into the 2D flap cutting guide based on its curvature and stretchability. The guide was then printed out using a 3D printer. The PANSOFOS study used RFFF in reconstruction for their patient, while the ALTF was used in our patients. Only one case was reported in their study. We compared the clinical outcome of 15 patients with oral and oropharyngeal SCC who underwent CARST and 15 patients who received the traditional reconstruction. The PANSOFOS study estimated possible shorter operative and hospital stay time and predicted lower costs for patients by comparing the outcome of their cases to the national report. Our study demonstrated significantly shorter operative and hospital stay time and a trend of fewer and milder postoperative complications.

There are several limitations of utilizing this technology in the reconstruction of soft tissue defects. The thickness of skin varies in different patients which in turn may influence the needed size of the skin flap. We need to improve the technology so that it can adjust the flap size based on skin thickness of the patient. We also need to enable it with 3D simulation capability so that it can ensure the reconstruction precisely matches the shape and volume of original soft tissue. In addition to measures of blood loss, operative and hospital stay times and flap utilization rate, more objective approaches to evaluate the impact of CARST and other similar technologies on oncological, functional, and cosmetic outcomes are needed.

## Conclusion

The novel CARST technology is safer and more efficient in the reconstruction of soft tissue defects following the resection of oral and oropharyngeal SCC. There is great potential for broader applications and better benefits with further improvement of the technology.

## Supplementary Information

**Additional file 1: Figure S1**: Enhanced CT scan of the tumor in the oral cavity. A. Upward to tongue back. B. Downward to ventral tongue and mouth floor. C. Inward beyond the midline to contralateral side. D. Outward to the right edge of the tongue. E. Forward to 1/3 of the tongue body. F. Backward to right tongue base. **Figure S2**: Appearance and tongue before and after the operation. A-D. Before the operation. E-H. After the operation. **Figure S3**: One-month post-operative CT scan. (A) Reconstructed tongue back, (B) Reconstructed tongue body, (C) Retained left tongue base, (D) Reconstructed mouth floor and flap pedicle, (E) Flap vessel, (F) Implanted vascular stapler.

## Data Availability

Data are available upon reasonable request to the corresponding author.

## Abbreviations

ALTF: Anterolateral thigh flap
CARST: Computer-aided reconstruction of soft tissue
ICU: Intensive care unit
PANSOFOS: Personalised pAtient-specific plaNning of SOFt tissue reconstruction
RFFF: Radial forearm free flap
SCC: Squamous cell carcinoma
