# Supplementary material for: Novel computer-aided reconstruction of soft tissue defects following resection of oral and oropharyngeal squamous cell carcinoma
Source: World J Surg Oncol. 2022 Jun 13;20:196. doi: 10.1186/s12957-022-02654-7 (PMC9195432; doi:10.1186/s12957-022-02654-7)

Supplemental data

Figure S1: Enhanced CT scan of the tumor in the oral cavity. A. Upward to tongue back. B. Downward to ventral tongue and mouth floor. C. Inward beyond the midline to contralateral side. D. Outward to the right edge of the tongue. E. Forward to 1/3 of the tongue body. F. Backward to right tongue base.


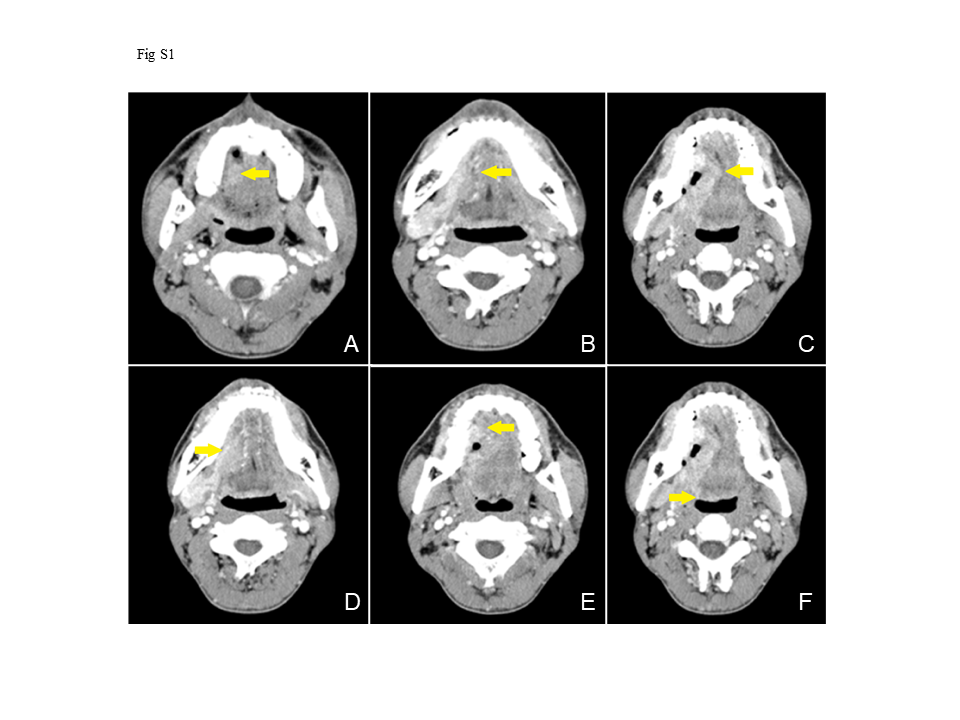


# Figure S2: Appearance and tongue before and after the operation. A-D. Before the operation. E-H. After the operation.

#
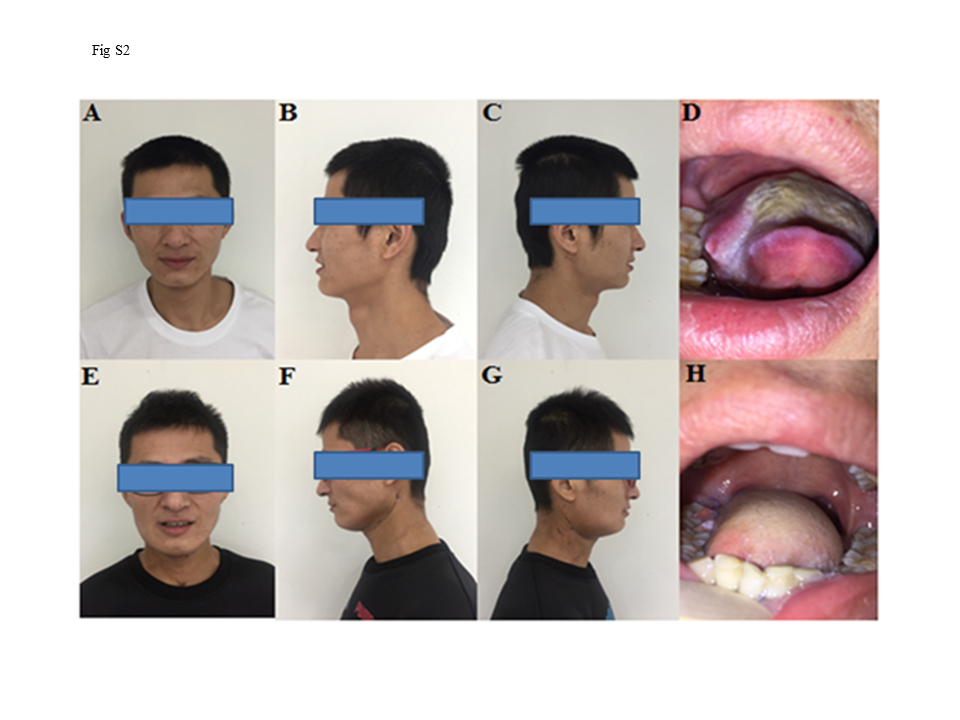


# Figure S3: One-month post-operative CT scan. (A) Reconstructed tongue back, (B) Reconstructed tongue body, (C) Retained left tongue base, (D) Reconstructed mouth floor and flap pedicle, (E) Flap vessel, (F) Implanted vascular stapler.


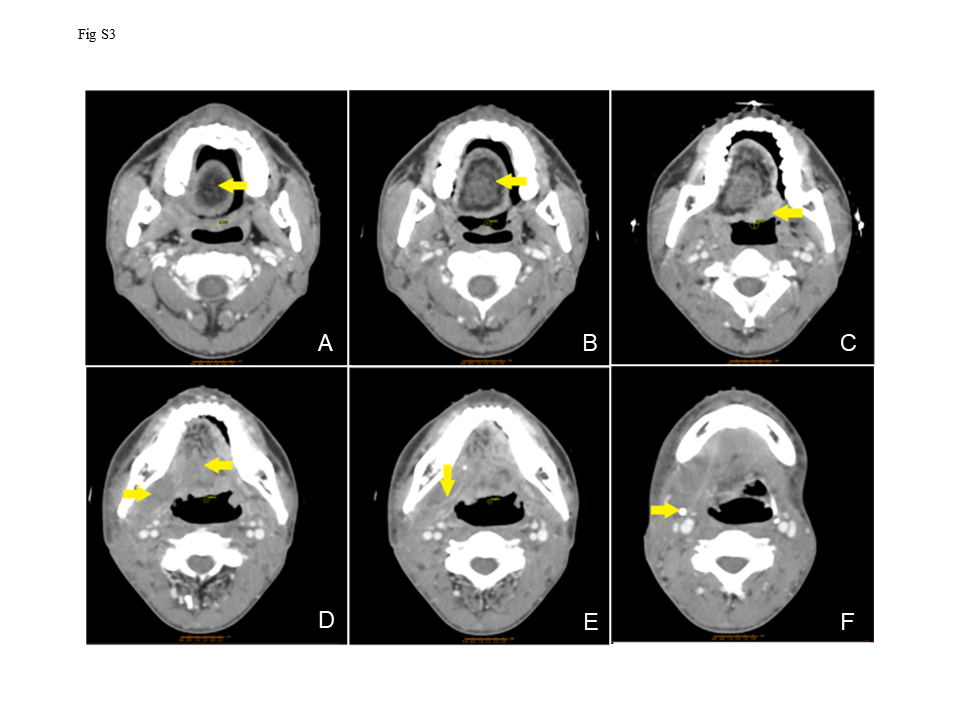

Supplement: Supplementary file 1 — Additional file 1: Figure S1: Enhanced CT scan of the tumor in the oral cavity. A. Upward to tongue back. B. Downward to ventral tongue and mouth floor. C. Inward beyond the midline to contralateral side. D. Outward to the right edge of the tongue. E. Forward to 1/3 of the tongue body. F. Backward to right tongue base. Figure S2: Appearance and tongue before and after the operation. A-D. Before the operation. E-H. After the operation. Figure S3: One-month post-operative CT scan. (A) Reconstructed tongue back, (B) Reconstructed tongue body, (C) Retained left tongue base, (D) Reconstructed mouth floor and flap pedicle, (E) Flap vessel, (F) Implanted vascular stapler. [file 12957_2022_2654_MOESM1_ESM.docx]
